# End-to-end automatic differentiation of the coronavirus disease 2019 (COVID-19) from viral pneumonia based on chest CT

**DOI:** 10.1007/s00259-020-04929-1

**Published:** 2020-06-22

**Authors:** Jiangdian Song, Hongmei Wang, Yuchan Liu, Wenqing Wu, Gang Dai, Zongshan Wu, Puhe Zhu, Wei Zhang, Kristen W. Yeom, Kexue Deng

**Affiliations:** 1 College of Medical Informatics, China Medical University, Shenyang, Liaoning 110122 People’s Republic of China; 2 School of Medicine, Department of Radiology, Stanford University, 1201 Welch Rd, Lucas Center, Palo Alto, CA 94305 USA; 3 Department of Radiology, Division of Life Sciences and Medicine, The First Affiliated Hospital of University of Science and Technology of China, No. 17, Lujiang Road, Hefei, 230036 Anhui China; 4 Department of Radiology, Anhui Provincial Hospital Affiliated to Anhui Medical University, Hefei, Anhui China; 5 Department of Radiology, the Lu’an Affiliated Hospital, Anhui Medical University, Lu’an, Anhui China

**Keywords:** Coronavirus disease 2019 pneumonia, BigBiGAN, Artificial intelligence, Differentiation, Semantic features

## Abstract

**Purpose:**

In the absence of a virus nucleic acid real-time reverse transcriptase-polymerase chain reaction (RT-PCR) test and experienced radiologists, clinical diagnosis is challenging for viral pneumonia with clinical symptoms and CT signs similar to that of coronavirus disease 2019 (COVID-19). We developed an end-to-end automatic differentiation method based on CT images to identify COVID-19 pneumonia patients in real time.

**Methods:**

From January 18 to February 23, 2020, we conducted a retrospective study and enrolled 201 patients from two hospitals in China who underwent chest CT and RT-PCR tests, of which 98 patients tested positive for COVID-19 (118 males and 83 females, with an average age of 42 years). Patient CT images from one hospital were divided among training, validation and test datasets with an 80%:10%:10% ratio. An end-to-end representation learning method using a large-scale bi-directional generative adversarial network (BigBiGAN) architecture was designed to extract semantic features from the CT images. The semantic feature matrix was input for linear classifier construction. Patients from the other hospital were used for external validation. Differentiation accuracy was evaluated using a receiver operating characteristic curve.

**Results:**

Based on the 120-dimensional semantic features extracted by BigBiGAN from each image, the linear classifier results indicated that the area under the curve (AUC) in the training, validation and test datasets were 0.979, 0.968 and 0.972, respectively, with an average sensitivity of 92% and specificity of 91%. The AUC for external validation was 0.850, with a sensitivity of 80% and specificity of 75%. Publicly available architecture and computing resources were used throughout the study to ensure reproducibility.

**Conclusion:**

This study provides an efficient recognition method for coronavirus disease 2019 pneumonia, using an end-to-end design to implement targeted and effective isolation for the containment of this communicable disease.

## Introduction

Coronavirus disease 2019 (COVID-19) pneumonia infections continue to increase in China and worldwide. As of April 6, 2019, the number of COVID-19 pneumonia cases globally was 1,210,956, resulting in more than 67,500 deaths [1, 2]. The World Health Organization declared a global health emergency on January 30, 2020 [3] and characterized the spread of COVID-19 as a pandemic on March 11, 2020 [4].

The virus nucleic acid real-time reverse transcriptase-polymerase chain reaction (RT-PCR) test is the current standard diagnostic method for diagnosing COVID-19 pneumonia [5], although it has limitations such as its low production, severe conditions for proper implementation and the number of false negatives [6]. Chest CT has proven to be a credible auxiliary tool for the clinical diagnosis of COVID-19 pneumonia [7, 8]. In recent literature, typical radiological imaging of COVID-19 pneumonia has clearly demonstrated the destruction of pulmonary parenchyma, including interstitial inflammation and extensive consolidation [9–11].

While thoracic radiological evaluation has been recognized as the key to diagnosing suspected COVID-19 patients [12], this method presents its own challenges. Studies have proven that non-COVID-19 patients with community-acquired infections caused by agents such as *Streptococcus pneumoniae*, *Mycoplasma pneumoniae* and *Chlamydia pneumoniae* present with CT signs similar to those in COVID-19 patients [9, 10, 13]. Symptoms such as fever, cough and fatigue are not unique to COVID-19 pneumonia and are observed in other virus-infected pneumonia cases [14]. Additionally, clinical practice has demonstrated no abnormality in the CT images of some COVID-19 patients, thus increasing the difficulty of diagnosing new coronavirus pneumonia infections in patients [12]. Due to the rapid spread of COVID-19 pneumonia, RT-PCR tests may not be available for all suspected cases. Accurately diagnosing COVID-19 pneumonia in patients with clinical symptoms and CT signs through an easy-to-implement method would be useful, in order to adopt targeted and effective isolation.

Artificial intelligence has provided significant breakthroughs in medical image analysis [15], particularly through a large-scale bi-directional generative adversarial network (BigBiGAN), a state-of-the-art deep learning algorithm that recognizes high-level semantic features of images [16]. Via self-supervised learning of these image features, the traditional method of learning image details has reformed, which transformed image generation quality improvement into representation learning performance improvement [17]. BigBiGAN has achieved a top rank score for image generation and semantic information extraction [18].

This study proposed an end-to-end automatic differentiation method for COVID-19 pneumonia patients outside the Hubei province in China. CT images of the patients with clinical and radiological symptoms of COVID-19 pneumonia were used for the extraction of semantic features by the proposed method. The output semantic feature matrix was then used for linear classifier training to distinguish COVID-19 pneumonia in suspected patients in real time.

## Materials and methods

Our institutional review board waived written informed consent for this retrospective study. There are no conflicts of interest to declare. From January 18, 2020 until February 23, 2020, we enrolled 98 patients with confirmed COVID-19 pneumonia (positive), and 103 patients with confirmed non-COVID-19 pneumonia (negative) in our study from The First Affiliated Hospital of University of Science and Technology of China and The Lu’an affiliated hospital of Anhui Medical University in China.

### Patient population

Patients who complained of cough, chest pain, vomiting, fever, sputum (with or without blood), fatigue, dizziness and sore throat were included. The exclusion criteria were as follows: (1) patients with no CT scans or incomplete clinical data; (2) patients with inconsistent test results; (3) with regard to the COVID-19 negative group, in addition to the above-mentioned main complaints, patients were included only when chest CT scans indicated signs of COVID-19 pneumonia infection or had one of the related exposure histories defined in this study. Testing for COVID-19 was carried out via laboratory testing with real-time RT-PCR tests using respiratory secretions obtained by bronchoalveolar lavage, endotracheal aspirate, nasopharyngeal swab or oropharyngeal swab. For included patients, positive or negative test results for COVID-19 were obtained at least twice. The RT-PCR test kits used for the patients in this study were manufactured by Jiangsu Shuoshi Biotechnology Co., Ltd. (Taizhou, China), Huada Biotechnology Co., Ltd. (Wuhan, China).

### CT examination

All patients underwent chest CT scans. We tabulated the number of days between symptom onset and the date of the CT scans. The time from the patient’s symptom onset to admission for CT examination was defined as early (0–2 days), intermediate (3–5 days) or late (6–12 days). In addition to the basic demographics of the patients, the related exposure histories were also included, specifically, history of travel to Wuhan in the previous 14 days, history of contact with a confirmed COVID-19 patient and history of contact with a dense crowd. The relevant exposure history was selected as an inclusion criterion since these patients had a high-risk of COVID-19 infection in China during this period. All COVID-19 positive patients were classified as having mild, common, severe or critical illness, according to the National Health Commission of the People’s Republic of China, Diagnosis and treatment of COVID-19 pneumonia (Tentative Standard 7).

In the study population, 77 patients from The First Affiliated Hospital of University of Science and Technology of China were imaged using a CT slice of 1-mm thickness on a GE Revolution 256 scanner (GE Medical Systems, Waukesha, American), and 89 patients were imaged with a CT slice of 5-mm thickness on NeuViz 128 scanner (Neusoft, Shenyang, China), and the other 35 patients from The Lu’an affiliated hospital of Anhui Medical University in China were imaged with a CT slice of 5-mm thickness on a NeuViz 64 scanner (Neusoft, Shenyang, China). All CT images were reviewed by two cardiothoracic radiologists with more than 10 years of experience each (H. W., Z. W.). The two radiologists were responsible for reviewing any potential signs of COVID-19 in chest CT scans such as bilateral involvement, peripheral distribution, mixed ground-glass opacity, and consolidation and vascular thickening according to the previous reports [12, 19]. The CT slices with suspected appearances and the whole CT scan of patients without abnormal findings were used in this study. All the images were reviewed independently by the two radiologists, and final decisions were reached by consensus have been reported.

### Training details

The patients from The First Affiliated Hospital of University of Science and Technology of China (83 COVID-19 positive patients and 83 COVID-19 negative patients) were categorized into a training dataset, a validation dataset and a test dataset by a ratio of 80%:10%:10%. The CT images from the other hospital (15 COVID-19 positive patients and 20 COVID-19 negative patients) were used for additional external verification. All CT images used were derived from the PACS system without any pre-processing to meet the end-to-end design. 2D CT images of the enrolled patients were used for the training and the extraction of semantic features by the BigBiGAN model. The output semantic feature matrix was then used for linear classifier training to distinguish COVID-19 pneumonia in suspected patients in real time. The BigBiGAN model was downloaded from the open-access TensorFlow Hub: https://tfhub.dev/s?publisher=deepmind&q=bigbigan.

Based on the strategy used in BigBiGAN [16], the features were extracted as the input of a linear classifier when the loss of the BigBiGAN model was minimal in the last epoch. Therefore, a linear classifier was also used in our study. Further, two widely used non-linear classifiers, support vector machine (SVM) and *k*-nearest neighbour (KNN), were used for comparison.

In order to improve reproducibility of this study, the execution of our algorithm was conducted on the free Google Colaboratory computing resource provided by Google Cloud. All CT data of this study has been publicly available, please see the URL link at the end of this paper. In addition, all code and resources for this study are publicly accessible at https://github.com/MI-12/BigBIGAN-for-COVID-19.

### Clinical application

Three different radiologists in our local hospitals with 3, 5 and 10 years of radiological experience independently reviewed the test datasets in this study. First, they performed the diagnosis without the assistance of BigBiGAN. The prediction results of the BigBiGAN model were then provided to the three radiologists, and a second round of diagnosis was performed by the radiologists. The sensitivity and specificity of the two diagnoses from the radiologists were recorded.

### Statistical analysis

R language (version 3.4.3 (Vienna, Austria)) was used for linear classifier construction and the evaluation of the accuracy of COVID-19 differentiation. The receiver operating characteristic curve (ROC) was used to present the results of COVID-19 classification, and the area under the curve (AUC) with sensitivity and specificity were used to evaluate the accuracy of differentiation. Chi-square tests and ANOVA tests were used to evaluate the differences in demographics between the two groups. *P* < 0.05 (two-tailed) was considered statistically significant.

## Results

### Patients

**Fig. 1:**
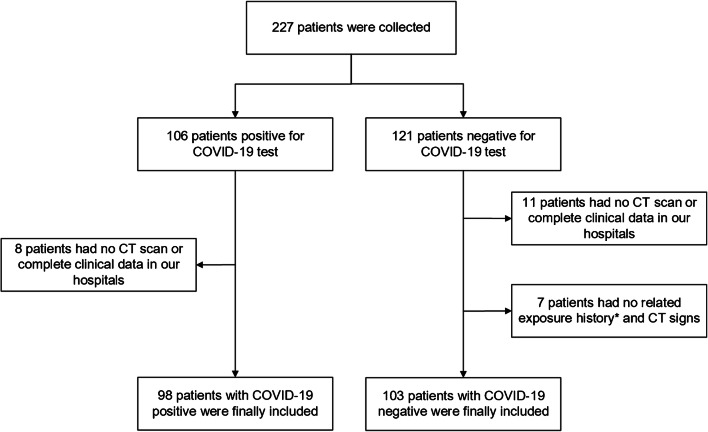
Patient enrolment for this study. *The related exposure history included the history of travel to Wuhan in the previous 14 days, history of contact with a confirmed COVID-19 patient and history of contact with a dense crowd

The COVID-19 positive group consisted of 60 males and 38 females, with an average age of 43 years. The COVID-19 negative group included 58 males and 45 females, with an average age of 39 years. Details of patient enrolment are shown in Fig. 1. No statistically significant differences in patient demographics are observed between the two groups (*P* > 0.05), except for the related exposure history (*P* < 0.05), as shown in Table 1.

**Table 1:** Demographics of patients enrolled in this study

Demographics	COVID-19 positive	COVID-19 negative	*P* value
Sex			0.479
Male	60	58	
Female	38	45	
Age median (SD)	43 (15.8)	39 (12.0)	0.064
Related exposure history			< 0.05
History to Wuhan	41	13	
Contact with infection	17	19	
Contact with dense crowd	40	58	
Classification			
Mild	6	/	
Common	65	/	
Severe	27	/	
Critical illness	0	/	
Basic disease (yes)	35	25	0.076

*COVID-19* coronavirus disease 2019, *SD* standard deviation

**Fig. 2:**
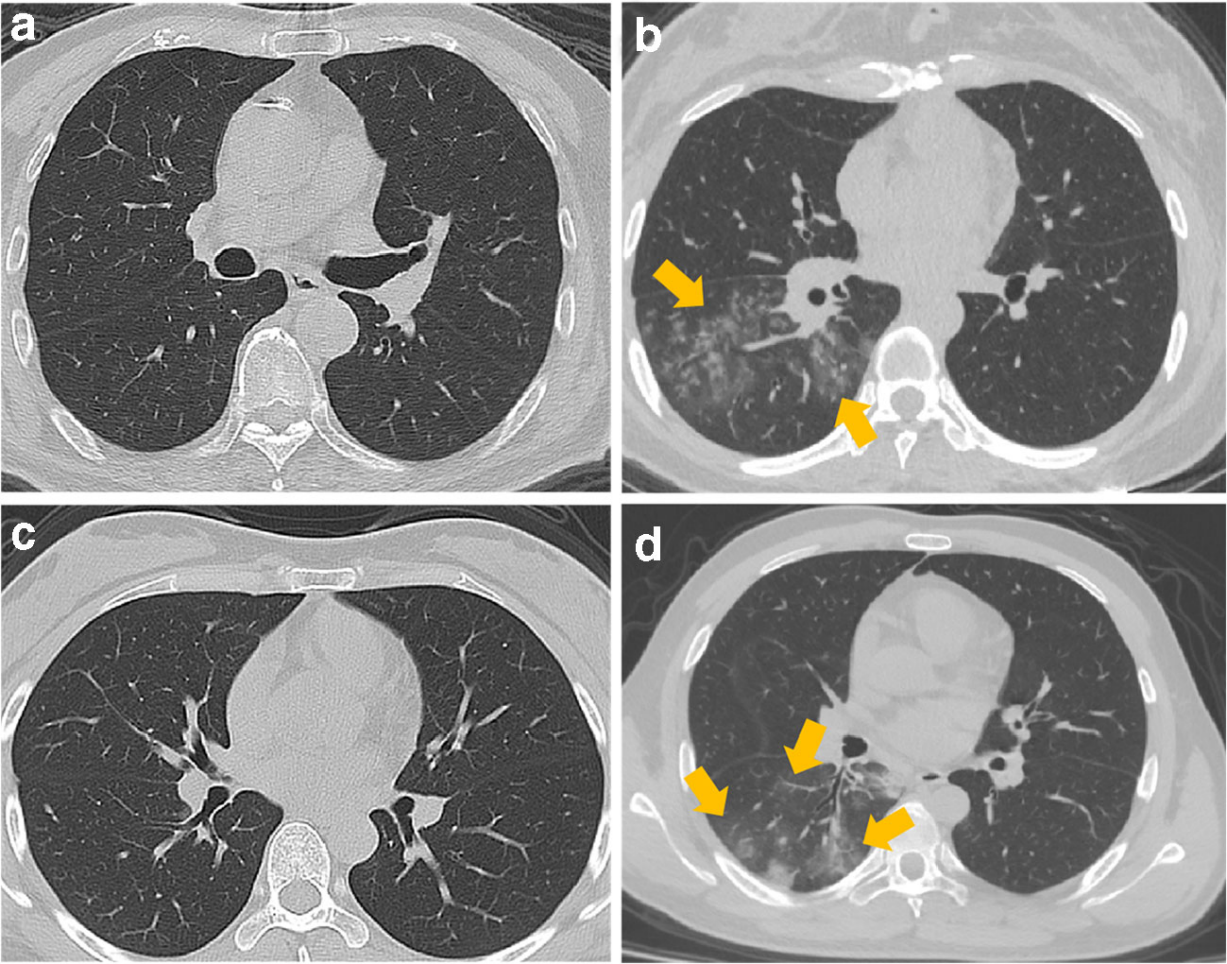
CT images of the coronavirus disease 2019 (COVID-19 negative pneumonia patients (**a**, **b**) and COVID-19 positive pneumonia patients (**c**, **d**)). **a** No abnormal findings on a CT of an 83-year-old male with a dry cough for 3 days and close contact with a COVID-19 confirmed patient for half a month; **b** flaky density shadows with multiple patches distributed in the lower lobe of the right lung of a 33-year-old female with the history of travel to Wuhan in the previous 14 days, and fever and cough for 5 days, and confirmed with mycoplasma pneumonia; **c** no abnormal findings on a CT scan of a 29-year-old female with the history of travel to Wuhan in the previous 14 days, and low fever and fatigue for 4 days, confirmed with COVID-19 positive; **d** flaky density shadows with multiple patches distributed appear in the lower lobe of the right lung of a 29-year-old male with fever and cough for 9 days, confirmed with COVID-19 positive

Cough and fever were the chief complaints and were observed in 97 (99%) of the individuals in the COVID-19 positive group and 93 (90%) of those in the COVID-19 negative group. Considering the time from the patient’s symptom onset to the admission for CT examination, there were 17 (17%) individuals classified as early, 36 (37%) as intermediate and 45 (46%) as late in the COVID-19 positive group, while the COVID-19 negative group had 34 (33%) of individuals classified as early, 36 (35%) as intermediate and 33 (32%) as late. The average number of RT-PCR tests in the COVID-19 negative group was 2.3 (all negative) and that of the COVID-19 positive group was 2.5 (all positive). All COVID-19 negative patients had community-acquired pneumonia infections and were admitted during the COVID-19 outbreak, and 91 (88%) cases had similar CT appearances to COVID-19 pneumonia after radiologists reviewed the CT scans. Among all patients, 10 patients with confirmed COVID-19 positive had no obvious CT abnormalities, while 12 patients in the COVID-19 negative group had related exposure history despite no obvious CT abnormalities. The CT images with and without obvious CT appearances of COVID-19 pneumonia in the two groups are shown in Fig. 2.

### Accuracy evaluation

**Fig. 3:**
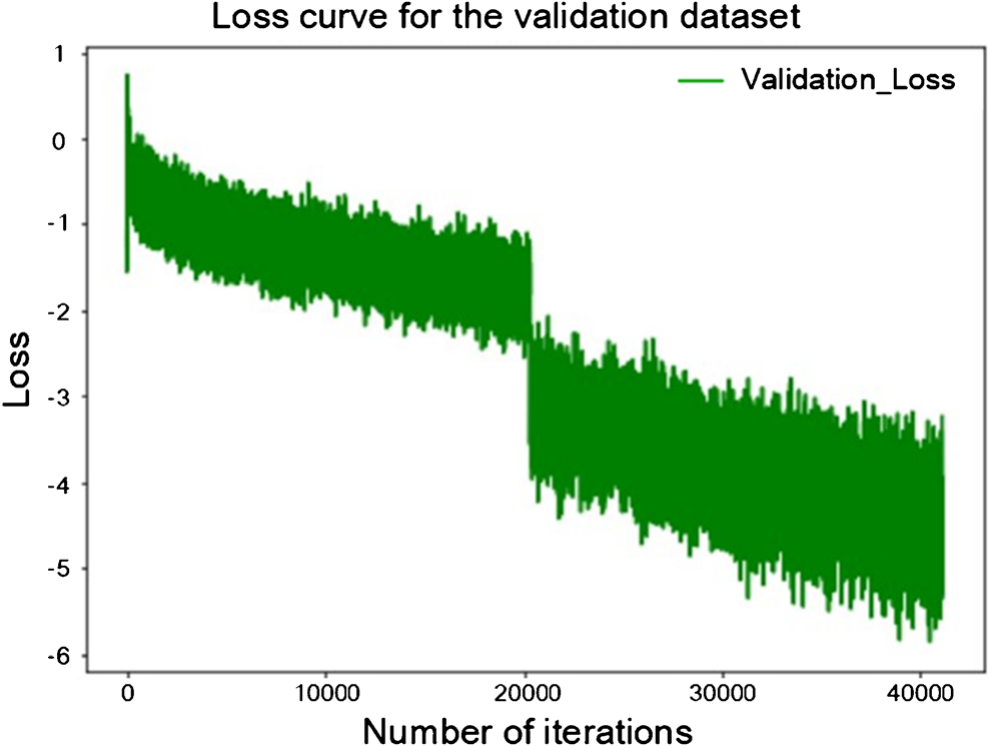
The loss curve of the validation dataset during the training of the BigBiGAN architecture in this study. When the algorithm was running to the 60th epoch, the cloud server computing resources provided by Google were exhausted. Due to rental time limitation, an “interrupt” of loss curve occurred when the cloud server was reconnected to continue execution

**Fig. 4:**
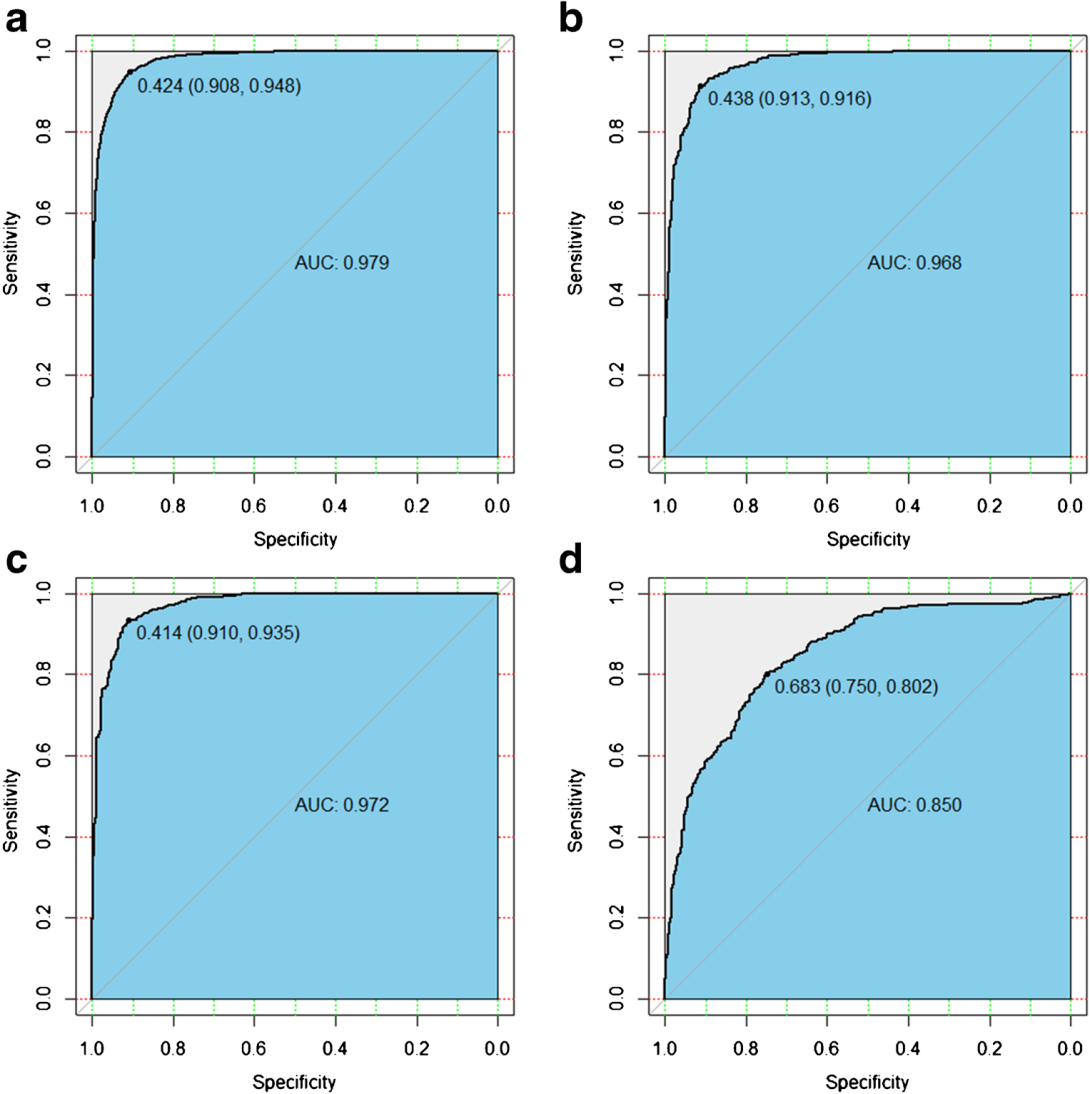
The receiver operating characteristic (ROC) curves of the training dataset (**a**), validation dataset (**b**), test dataset (**c**) and external validation dataset (**d**). The area under the curve and the cut-off value with specificity and sensitivity were presented in each ROC curve

The epoch of BigBiGAN execution was set to 120, with a batch size of 16. The loss curve of BigBiGAN is shown in Fig. 3. Based on the 120-dimensional imaging semantic features extracted from each image, a linear classification was constructed by using the “lm” package in R. Details of the code and semantic feature matrix can be found at https://github.com/MI-12/BigBIGAN-for-COVID-19. The AUCs of the training dataset, validation dataset, and test dataset were 0.979, 0.968 and 0.972, respectively, with an average sensitivity of 92% and specificity of 91%, as shown in Figs. 4a-c.

Based on the external validation of the CT images obtained through different data scanning machines from the other hospital, the AUC of 0.850 with a sensitivity of 80% and specificity of 75% is obtained, as shown in Table 2 and Fig. 4d. The time consumption of the analysis for one batch size was 0.25 s in this study.

**Table 2:** The sensitivity and specificity of the differentiation of COVID-19 pneumonia by radiologists and the method in this study. R1 to R7 represent the three Chinese radiologists and four US radiologists reported in reference [20]

	R1 (%)	R2 (%)	R3 (%)	R4 (%)	R5 (%)	R6 (%)	R7 (%)	Ours
Sensitivity	80	67	97	93	83	73	70	(12/15) 80%
Specificity	100	93	7	100	93	93	100	(15/20) 75%

When using SVM as the classifier, 1.000, 0.517, 0.531 and 0.500 of AUC were obtained on the training dataset, validation dataset, test dataset and external validation dataset, respectively. For KNN classifier, 0.997, 0.998 and 0.734 of AUC were obtained on the validation dataset, test dataset and external validation dataset, respectively.

### Clinical application

The average sensitivity and specificity of diagnosis by the three radiologists without the assistance of BigBiGAN were 77% and 75%, respectively. When the prediction result of BigBiGAN was provided as a reference, the average sensitivity and specificity improved to 85% and 88%. The results are presented in Table 3.

**Table 3:** The sensitivity and specificity of diagnosis of the test datasets by the three radiologists with and without the assistance of the BigBiGAN. R1, R2 and R3 indicate the three radiologists in our local hospitals

	Without BigBiGAN		With BigBiGAN	
	Sensitivity (%)	Specificity (%)	Sensitivity (%)	Specificity (%)
R1	70	72	78	83
R2	92	87	95	92
R3	69	66	82	89
Average	77	75	85	88

## Discussion

For the diagnosis of severe coronavirus disease 2019 pneumonia, we proposed an end-to-end automatic differentiation method based on CT images. Representation learning through state-of-the-art BigBiGAN framework was conducted for semantic feature extraction from CT images of patients testing positive and negative for COVID-19. Based on the linear classifier constructed by the semantic feature matrix, our study demonstrated that the proposed approach accurately detects COVID-19 pneumonia infections in a population with similar CT appearances, with a sensitivity of 80% and specificity of 75% on the external validation dataset. In the event that nucleic acid detection and radiological experts are not available, this method can be used as a credible adjuvant clinical tool to conduct real-time screening of suspected COVID-19 patients, to guide the implementation of targeted isolation to avoid further transmission.

Although the RT-PCR test is the standard for the clinical diagnosis of COVID-19 infections, several limitations apply to its application [5]. Radiographic chest CT imaging is considered an effective auxiliary diagnostic method [7, 21]. However, it becomes difficult for radiologists to accurately distinguish COVID-19 patients from patients with viral pneumonia infections caused by *S. pneumoniae*, *M. pneumoniae* and *C. pneumoniae*, when accompanied by CT findings similar to those of COVID-19 [19]. Our findings provide a reliable auxiliary solution to these challenges to improve the radiologists’ diagnostic accuracy of COVID-19. The average sensitivity of radiologists’ diagnosis improved from 77 to 85%, and the specificity improved from 75 to 88% with the assistance of our approach.

Based on the comparison experiment with non-linear classifiers, we found that severe overfitting will be produced by SVM when using the features extracted by BigBiGAN. Although overfitting was partially relieved by KNN, the prediction accuracy was still lower than that of the linear classifier (0.734 vs. 0.850), and the training time was 15 times longer than a linear classifier. Therefore, for the semantic features extracted by BigBiGAN, the performance of classification using a linear classifier will be better than that of a non-linear classifier, and the running time will be significantly reduced.

The results from this study indicated that the difference in CT characteristics between patients testing COVID-19 positive and other viral pneumonia patients could be decoded by a state-of-the-art artificial intelligence technique. Although the CT findings of other viral pneumonia infections were similar to those of COVID-19 pneumonia, the self-supervised learning method of BigBiGAN distinguishes the two groups of patients based on the level of image semantic knowledge. Existing evidence shows that the CT signs of COVID-19 pneumonia are bilateral involvement, peripheral distribution, mixed ground-glass opacity and consolidation, and vascular thickening [22]. Thus, traditional methods may not be suitable for this issue because of the following reasons. First, if large bilateral involvement of the lungs occurs, it is difficult to accurately outline the area of interest on CT image. Second, there are some patients with COVID-19 pneumonia who have no obvious abnormal CT signs [12]. The end-to-end artificial intelligence analysis technology can help avoid the shortcomings of traditional analytic methods for this issue. Specifically, the BigBiGAN design does not concern itself with the image details but extracts the abstract frame elements that make up these details; that is, the semantic knowledge of the content expressed by the image was decoded and quantified. The entire CT image was used as the input, and the semantic features of bilateral involvement, peripheral distribution, ground-glass opacity and vascular thickening in COVID-19 pneumonia were identified by BigBiGAN. Meanwhile, with the semantic features extracted from the other viral pneumonia CT images, the difference between the CT finding of COVID-19 pneumonia and other viral-infected pneumonias can be quantified and evaluated. By using the external validation dataset to reduce bias, the results of our study indicated that based on the encoding of the semantic features, a sensitivity of 80% and specificity of 75% were achieved on the external validation dataset for COVID-19 pneumonia differentiation. Compared with the values of average sensitivity and specificity values of 80% and 83%, respectively, reported for the differentiation of COVID-19 from viral pneumonia using chest CT scans [20], our external validation results and the clinical application experiment indicated that the proposed approach could be used as a reliable clinical assistance for COVID-19 pneumonia differentiation.

Results indicated that our study could distinguish the patients who had no obvious CT abnormalities between COVID-19 positive and COVID-19 negative patients, as shown in Fig. 2. One potential reason is that although the COVID-19 features such as bilateral involvement, peripheral distribution, mixed ground-glass opacity or consolidation or vascular thickening were not observed with the naked eye, the subtle changes of COVID-19 had, nonetheless, been occurring in the lungs. These subtle changes were captured and expressed as differences in semantic features through the various filters in the digital deep learning network. Although other deep learning algorithms have been proposed for the screening of COVID-19 using CT images, this study had advantages when compared with other studies [23, 24]. More than 1000 COVID-19 positive patients were used in a recent study to propose a deep learning framework to distinguish COVID-19 positive patients and other patients, but the ratio of COVID-19 to other patients on the training dataset was 1:6.5 [25]. The mismatch in data volume limited the significance of the study [26]. Our end-to-end study design eliminated complex image pre-processing such as the cube selection of traditional convolutional neural networks [23], thus increasing the reproducibility of this study. Clinicians, researchers and patients from different backgrounds can independently test their own data using the free computing resources described in the present study. Additionally, the use of the external validation dataset further ensures the robustness and credibility of our proposed method for differentiating COVID-19 pneumonia infections from other pneumonia infections, which was not mentioned in previous studies [24]. Finally, in this study, all experimental resources have been made publicly accessible for future research, to facilitate its reproducibility.

The strategy of using BigBiGAN and a linear classifier is feasible for other lung lesion–related diseases, such as lung nodules or lung cancer. The method proposed in this study can be considered as first extracting the semantic features on CT images and then supervised learning of the classification of the semantic features. Therefore, the different expressions of lung-related diseases on CT images also could be effectively captured and analysed by this approach. Compared with current imaging feature–based analysis methods, for example, radiomics [27], the advantage of BigBiGAN is that the algorithm does not consider the detailed texture of the region of interest but captures the difference in the semantic information patterns expressed by different images. The performance of BigBiGAN on image semantic recognition has been shown [17], which further demonstrates the potential for BigBiGAN to be applied to the diagnosis of other lung lesion diseases.

The limitations of this study are as follows. First, a decrease in classification accuracy was found on the external validation dataset. The primary reason may be the training sample size. More patients from multiple centres should be enrolled in future studies to improve the accuracy and generalize ability of the proposed model in differentiating COVID-19 from viral pneumonia. Differences in CT image acquisition, such as scanning parameters and reconstruction algorithms, will also produce a variation of image semantic feature extraction by BigBiGAN. Therefore, the diversity of training data should also be guaranteed in future research. A more robust model can be achieved through continuous validation by future researchers, using the open-access code of this study. In addition, the classifier that we used may have also affected the results. We used two other non-linear classifiers for comparison in this study, but both produced a certain reduction in prediction accuracy on the external validation dataset. Therefore, for each specific task, researchers should consider different classifiers to achieve optimized results. In addition, of all the enrolled patients, there were no COVID-19 patients with a critical illness. Future studies with larger sample sizes for study populations that include critically ill patients will provide a more comprehensive evaluation of COVID-19 pneumonia patients.

In conclusion, the present study provides a useful and effective recognition method for COVID-19 pneumonia using an end-to-end design. In the case that the RT-PCR tests and radiological experts are not available, this method can screen populations with suspected COVID-19 in real time, in order to implement targeted and effective isolation for the containment of this communicable disease.

## Data Availability

https://data.mendeley.com/datasets/kk6y7nnbfs/1
